# A perspective on using experiment and theory to identify design principles in dye-sensitized solar cells

**DOI:** 10.1080/14686996.2018.1492858

**Published:** 2018-08-23

**Authors:** Peter J. Holliman, Christopher Kershaw, Arthur Connell, Eurig W. Jones, Robert Hobbs, Rosie Anthony, Leo Furnell, James McGettrick, Dawn Geatches, Sebastian Metz

**Affiliations:** a College of Engineering, Swansea University, Bay Campus, Swansea, UK; b Scientific Computing Department, STFC Daresbury Laboratory, Daresbury, Warrington, UK

**Keywords:** DSC, Surface engineering, review, half-squaraine dyes, computer modelling, 50 Energy Materials, 101 Self-assembly / Self-organized materials, 209 Solar cell / Photovoltaics

## Abstract

Dye-sensitized solar cells (DSCs) have been the subject of wide-ranging studies for many years because of their potential for large-scale manufacturing using roll-to-roll processing allied to their use of earth abundant raw materials. Two main challenges exist for DSC devices to achieve this goal; uplifting device efficiency from the 12 to 14% currently achieved for laboratory-scale ‘hero’ cells and replacement of the widely-used liquid electrolytes which can limit device lifetimes. To increase device efficiency requires optimized dye injection and regeneration, most likely from multiple dyes while replacement of liquid electrolytes requires solid charge transporters (most likely hole transport materials – HTMs). While theoretical and experimental work have both been widely applied to different aspects of DSC research, these approaches are most effective when working in tandem. In this context, this perspective paper considers the key parameters which influence electron transfer processes in DSC devices using one or more dye molecules and how modelling and experimental approaches can work together to optimize electron injection and dye regeneration.

## Graphical Abstract

**Figure:**
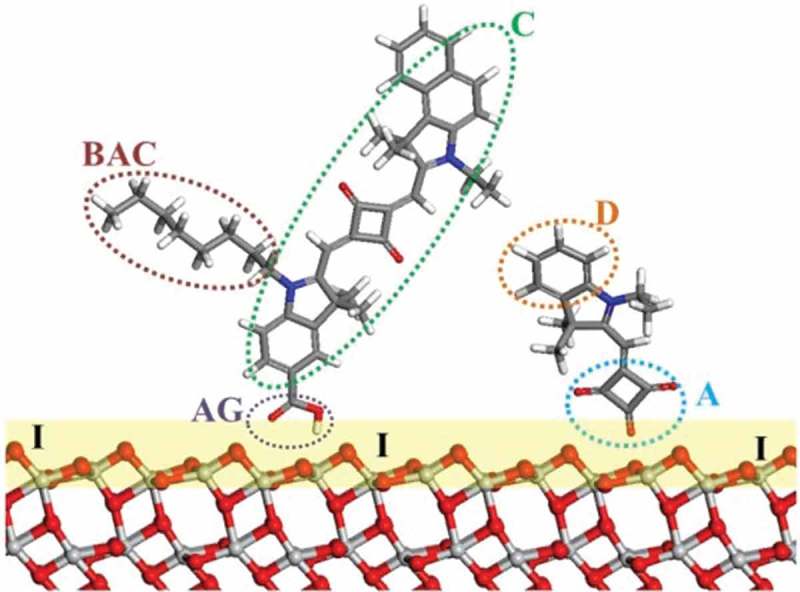
This paper provides a perspective that theory and experiment are best used in tandem to study DSC devices

## Introduction

Since their invention in the late 1980s [1] dye-sensitized solar cells (DSC devices) have developed to become one of the most promising forms of solar photovoltaic (PV) technology [2]. This is partly because they contain predominantly earth-abundant elements which are environmentally safe and they can be manufactured under ambient conditions using roll-to-roll (printable) techniques which are more cost efficient to scale than vacuum-based technologies.

DSC devices are fabricated using a dye/sensitizer which is chemisorbed onto a semi-conducting metal oxide photoanode (usually TiO_2_ or ZnO).

**Figure 1.**
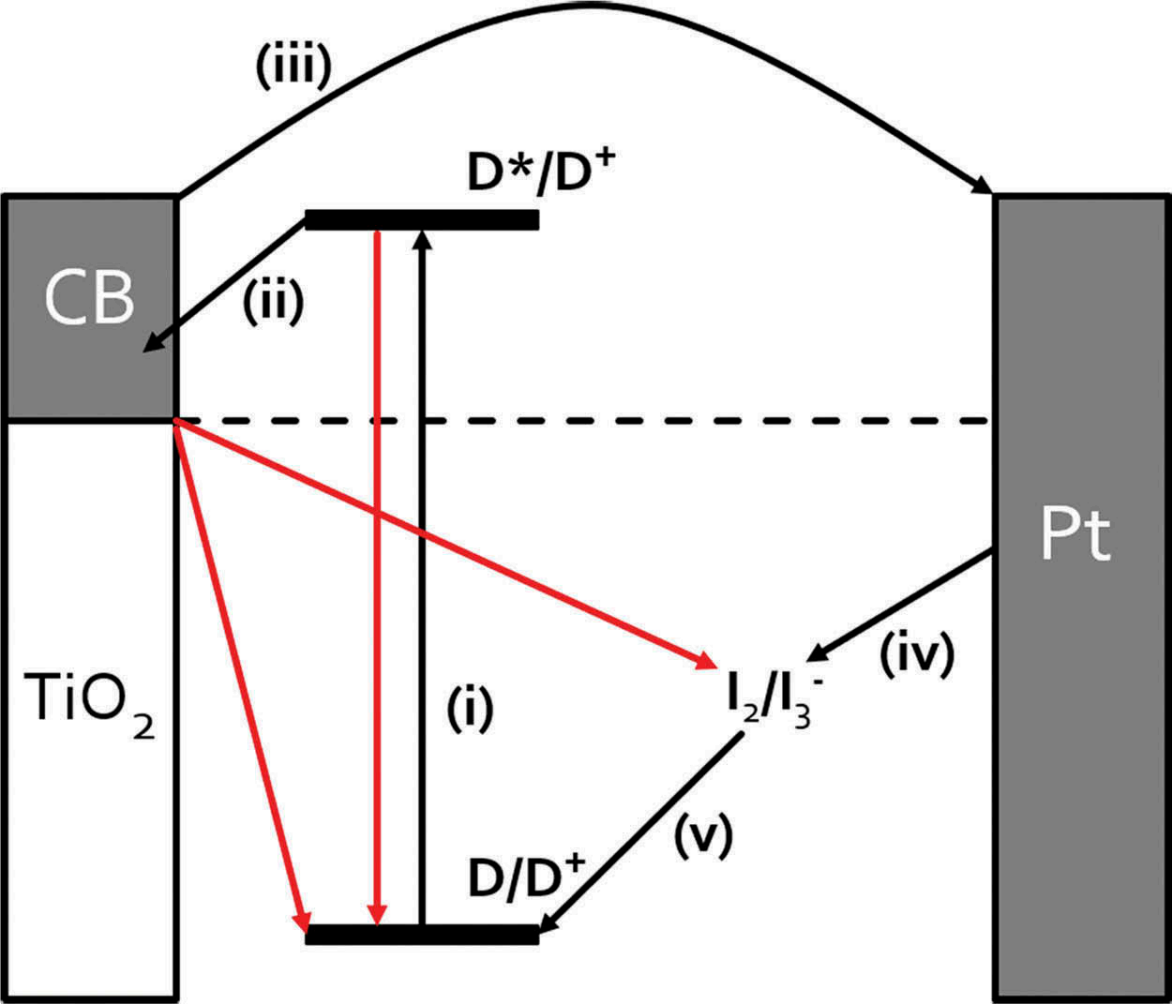
Schematic of DSC device showing key steps for device operation (in black) and competing processes (in red).

The metal oxide photoanode consists of pre-made nanoparticles which are then sintered onto a conducting substrate to form a mesoporous film. For liquid DSC devices, this dyed metal oxide photo-anode is sealed against a counter electrode and a liquid electrolyte containing a redox couple (usually I_3_^-^/I^-^) is added into the void. Figure 1 shows the main processes which take place during the operation of a liquid DSC device [3,4]. Absorption of light results in photoexcitation of the dye sensitizer (i) which promotes an electron from the highest occupied molecular orbital (HOMO) to the lowest unoccupied molecular orbital (LUMO), followed by injection into the conduction band (CB) of the metal oxide semi-conductor (ii). Photogenerated electrons travel through the metal oxide nanoparticles to the photoanode which can be connected to an external circuit (iii). The sensitizer is regenerated by electron transfer from the redox couple of the electrolyte (iv). The oxidized electrolyte is then regenerated by electron capture from the redox couple of the electrolyte (v). Regeneration of the oxidized electrolyte is achieved by recapturing electrons from the counter electrode (vi). The circuit is completed by electrons returning to the cell through the counter electrode which is a conducting substrate typically coated with platinum or carbon nanoparticles. During device operation, processes (i–v) cause the CB of TiO_2_ to fill which increases its conductivity and raises the TiO_2_ Fermi level which increases the device open circuit voltage (*V*_oc_). However, the electrons can also undergo back reactions (e.g. the TiO_2_ or excited dye can transfer electrons back to the oxidized redox couple) which lowers the Fermi level and *V*_oc_ [3]. In practice, TiO_2_ films which are thinner than the electron diffusion length are favoured because the electrons reach the collection electrode before they can take part in any back reactions.

The overall efficiency of DSC devices is determined by the *V*_oc_, the short-circuit current density (*J*_sc_) and the fill factor (FF).

$$\eta =V_{oc}\times J_{sc}\times FF$$

where *V*_oc_ is the voltage difference between the redox couple and the Fermi level of the TiO_2_. To optimize *V*_oc_, the TiO_2_ Fermi level should be as high as possible while the redox couple energy level should be as low as possible. However, in a single-dye DSC device, the *V*_oc_ is limited because to substantially raising the TiO_2_ Fermi level requires a dye with a very large HOMO–LUMO gap. In turn, this would mean that only high energy photons would be able to generate photocurrent and, because there are fewer of these in the solar spectrum, this would limit light harvesting and *J*_sc_. This is part of the Shockley–Queisser limit for single-junction solar cells [4].

In practice, liquid DSC devices using iodine-based electrolytes are typically limited to *V*_oc_ of ≈ 0.8 V because the conversion between I_3_^−^ and I^−^ takes place through several steps with various energy states [3]. Each of these steps, and the regeneration of the dye itself, requires an over-potential (≈ 0.2 eV) to efficiently drive the process. By comparison, one electron change redox couples (e.g. based on Co complexes) and solid hole transport materials (HTMs) can achieve higher voltages (i.e. ≈ 1.0 V) because fewer steps are involved and overpotential is lost during each step [3]. This also means that the position of the dye energy levels is key to optimizing device voltage; the LUMO needs to be ≈ 0.2 eV above the metal oxide CB and the redox couple [4]. The device *J*_sc_ reflects the light harvesting and electron injection efficiency of the dye sensitizer. The more light which is absorbed and the faster electrons are injected from the dye excited states, the higher the *J*_sc_. In practice, *J*_sc_ of 18–20 mA cm^−2^ are relatively easy to achieve. And finally, the FF is most heavily influenced by the series resistance of the device. During operation, the regeneration of the excited state dye is usually the slowest process and so this usually influences the FF most strongly.

This review considers experimental data related to dye developments which have traditionally provided the details of the mechanisms of DSC devices. However, in the last 10–20 years the developments in computational modelling have produced a toolbox of techniques to probe chemical and physical properties of condensed matter providing levels of detail that are not easily accessible experimentally. The choice of tool depends primarily on the length- and time- scale explored. For example, to probe (time-independent) chemical reactivity requires electronic structure (quantum mechanical – QM) techniques such as density functional theory (DFT) whereas probing excited states requires more sophisticated electronic structure methods such as time dependent (TD-)DFT or many body perturbation theory (MBPT). By comparison, to simulate dynamic processes occurring over nanoseconds, where chemical reactivity (i.e. electron transfer) is irrelevant, the tool of choice would be molecular dynamics (MD).

Increasing time and length scales necessitate a decrease of atomistic detail to counter the increasing computational demands. For example, while DFT is an *ab initio* method that models electrons within atoms, MD employs empirical data to model atoms and the interactions between them but ignores electrons while quantum mechanics/molecular mechanics (QM/MM) uses a hybrid mix of electronic structure methods to explore a ‘small’ region of reactivity embedded within a larger, non-chemically reactive system. Moving to longer length-scales, mesoscale methods (such as coarse-graining) ignore atomistic detail, encapsulating whole or parts of molecules within beads, to enable the exploration of phase properties. These methods comprise a suite of tools in a multi-scale tool box which have been used to explore multi-component materials such as DSC devices.

Previous reviews (and the references within) of the development of transition metal or organic dye sensitizers, provide a good outline of the different components of DSC devices [5,6]. In the following sections we first describe the components of DSCs from the experimental perspective. This is then followed by a discussion of the challenges involved in atomistic, computational modelling of these complex materials. These sections include developments and the current challenges faced in their fabrication, characterization, and measurement.

## Experimental and theoretical methods

### Experimental methods

The synthesis of new sub-components for DSC devices typically involves multi-step syntheses often requiring labour intensive purification (e.g. column chromatography). This means that great care must be taken when designing new materials. In this context, theoretical modelling can provide valuable insights (e.g. predicting HOMO–LUMO levels) to minimize synthetic time by helping to identify desirable target dye molecules [7,8].

Testing new materials in devices is challenging because DSC devices contain many components arranged in series in an electrical circuit [9]. Thus, if any one component is not optimized then the whole device efficiency suffers (even if it is not the component being tested). In practice, this means that multiple devices must be manufactured alongside control devices which is time consuming. In addition, as the device layers become thinner (for example, in solid state DSC devices) then the need for dust-free manufacturing environments becomes more important.

In addition, new components are typically tested on laboratory-scale devices (≤ 1 cm^2^) soon after manufacturing. However, for any new components and the related devices to be suitable for commercial use, they must have extended lifetime (≈ 5 years for indoor use and ≈ 25 years for outdoor deployment). So, the next level of device testing is typically accelerated lifetime testing and device scaling. However, even with accelerated testing, lifetime studies of PV devices require months of exposure for each iteration [9]. Ultimately, what this emphasizes is that combining theoretical and experimental approaches to the design and understanding of solar cell components can reduce the number of materials which need to be synthesized and tested which, in turn, significantly accelerates research progress.

### Theoretical parameters and methods

Building any atomistic model requires undertaking a series of steps; from first understanding the composition of the material, determining the size of model, deciding the properties of interest, etc. These decisions are not independent of one another. For example, one of the least computationally expensive methods to obtain excited state data is TD-DFT, which determines the number of atoms it is feasible to model given the resources available. On the other hand, exploring dye orientation can be addressed by force-field based MD methods.

Within each of these decisions there are more to make depending on the modelling method. For example, DFT requires inputs such as: the basis set, the type of pseudopotential, the exchange-correlation functional, and possibly the Hubbard value. While their description is beyond the scope of this review, there are many versions of both from which to choose and some studies focus solely on exploring these options [10,11].

When probing excited states there are several options available such as TD-DFT, coupled cluster, multi-reference perturbation theory, real time and frequency domains; see [12,13] and the references within for more details.

Although measuring excitation energies experimentally is fairly straightforward, reproducing these values theoretically is both computationally expensive and challenging. Excited state modelling of dyes in solution can be done using a polarizable continuum model (PCM) [13,14], but this model is not applicable to dyes adsorbed at surfaces. In the latter case the size of the system should be consistent with the computational resources available.

Other issues arise such as artefacts of model size, for example, when simulating a slab of TiO_2_ and adsorbate at its surface, care must be taken to avoid dipole polarization across the slab. One way to ameliorate this is to use a symmetric model – including adsorbates at both surfaces of the slab[15].

**Figure 2.**
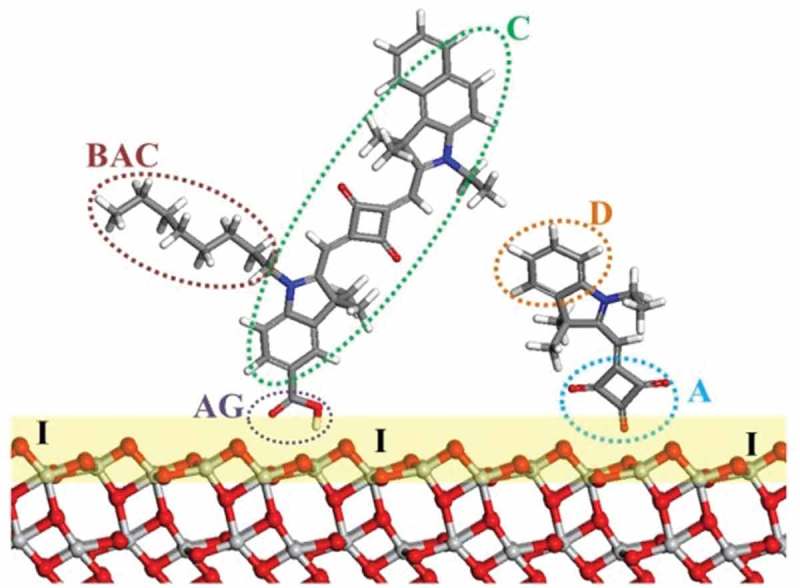
Schematic of DSC components illustrating dye co-sensitization: C, dye chromophore; BAC, bulky alkyl chain; D, donor; A, acceptor; AG, anchoring group; I, TiO_2_/dye interface. Red, O; light grey, Ti; blue, N; grey, C; white, H.

In the end the choice of modelling method and its parameters depends on the property and components of interest. Previous theoretical reviews (and the references within) of the development of transition metal mesoporous photosensitizers, provide a good outline of the different components of DSC [5,6]. A schematic description of the adsorbed dye molecules is shown in Figure 2.

## TiO_2_ photoanodes

### Experiment

The TiO_2_ photoanode is actually a mesoporous film consisting of anatase TiO_2_ nanoparticles (typically ≈20 nm diameter). Before mixing these nanoparticles into a printable colloid to be used in device manufacturing, they are hydrothermally treated to improve their crystallinity and minimize defects while maintaining their small particle size and high surface area. The TiO_2_ photoanode is fabricated by printing the colloid onto a conductive substrate followed by heating to sinter the particles together to ensure good mechanical strength and inter-particle connectivity to allow charge to pass through the mesoporous film to the underlying substrate [16]. While the films are typically heated to 450/500 °C for 30 min to 1 h, this can reduce the active surface area for dye sorption [16] so it is preferable to sinter films at as low a temperature as possible and for the shortest possible time. The colloid content (i.e. organic binders etc.) will change the sintering time and temperature required. At the same time, while 7–10 μm films are typical for liquid DSC devices, thinner films (≈ 2 μm) are preferable for solid state DSC devices because this helps with pore filling by the solid electrolyte. This inevitably impacts on dye loading with ≈ ⅓ of the dye molecules possible in the thinner films. This limits short circuit current for Ru-bipyridyl dyes such as N719 (ε ≈ 13,500 M^−1^ cm^−1^) [17] so it is necessary to use sensitizers with ≥ 3 times higher molar extinction coefficient which means organic dyes (e.g. half-squaraine with ε ≥ 100,000 M^−1^ cm^−1^) [7].

### Theory

From an experimental perspective, the detailed, nano-scale surface properties of the TiO_2_ electrode, beyond choosing anatase or rutile, can be difficult to study under realistic device conditions. But from an atomistic modelling perspective it is vital to understand its inherent properties. For example, as recorded by O’Regan and Durrant [18] on a TiO_2_ particle of 18 nm diameter, there are ~ 10,000 adsorption sites for H^+^ but are all of these sites equal? Does an adsorption site at the edge of the nano-particle have the same electronic environment as an adsorption site at the centre of the particle’s surface?

QM methods can realistically explore hundreds to thousands of atoms in a system using ‘reasonable’ computational time and resources, but this does require simplifying assumptions in order to model a representative sample of the TiO_2_ electrode. This initially involves modelling an isolated slab of TiO_2_ to determine the correct Miller plane, number of atomic layers and the required surface area [10]. The result is a model of a nano-scale portion of a TiO_2_ electrode if using a periodic QM code, or an angstrom-to-nano-scale sized cluster.

Macroscopic properties of metal oxides such as grain boundaries, lattice defects and impurities are also very important to DSC devices because they trap electrons and holes, and they are difficult to capture at the nano-scale. An overview of electron and hole trapping in metal oxides presents successful examples of models from the perfect lattice to structural defects and impurities, and emphasizes the importance of charge localization [19].

## Development in sensitizer/dyes

### Experiment

In DSC devices, the dye sensitizer is one of the key components for high power conversion efficiencies. Extending the spectral response of the sensitizer has been a focal point when trying to increase device performance. Attempts have been made to synthesize dyes with extended spectral response (panchromatic dyes) such as ‘black dye’ or ‘squaraines’. However, the achieved increases in *J*_sc_ have previously come at the expense of *V*_oc_ because the dye HOMO–LUMO gap is decreased in order to extend the absorption onset to capture lower energy (longer λ) photons [20].

Among the transition metal dye sensitizers studied, ruthenium-bypyridyl (Ru-bipy) complexes have been the most widely used sensitizers in DSC devices [3]. Examples of these dyes include C106 [21], N719 [22] and C101 [1,20]. Ru-bipy dyes are highly efficient at ca. 550 nm but their response drops dramatically between 650 and 700 nm [23]. Despite relatively simple synthesis, the purification of Ru-by dyes is complex making them challenging to upscale. In addition, Ru is expensive which makes Ru-bipy DSC dyes very expensive. The combination of these factors makes these dyes less desirable for the industrial production of DSC devices [24]. These problems have led to the development of many new organic dyes including triarylamines [25–27], coumarins [28], cyanines [29], indolines [30,31], squaraines [32–34], quinoxalines [35] and natural dyes [36]. These organic dyes are designed with a donor-ᴨ-linker-acceptor (D-π-A) structural arrangement, and absorb in the same region as Ru-by dyes. They are easier to synthesise, less demanding to purify, much cheaper and more environmentally friendly [23]. Organic dyes often have higher molar extinction coefficients (ε) than their inorganic counterparts which can be extremely useful. Dyes with higher ε can be used in smaller/thinner devices while absorbing just as much light. The use of thinner metal oxide photoanode films leads to decreased recombination losses and improved *V*_oc_ [37].

To date, liquid DSC devices have reached efficiencies of η > 14.7% [38] using a device co-sensitized with two dyes. The sensitizers used were ADEKA-1 which consists of a carbazole donor, an alkyl-functionalized oligothiophene linker and an alkoxy-silyl linker while the second dye is the triphenylamine (TPA)-based LEG4 dye which has a carboxylate (COOH) linker. The authors suggest that this combination of dyes work collaboratively to improve electron injection [21]. In addition, solid state DSC devices have recently reached 11% efficiency using the TPA dye Y123 and a mixture of Cu(I/II) bipyridines as the HTM [39].

To improve dye response further, given that the core structure chromophore is the part of a dye molecule responsible for light absorption, one approach to optimizing dyes is to maintain the core structure of the chromophore while modifying other parts of the dye molecule. One of the most recent and promising chromophores is the class of half-squaraine (HfSQ) dyes. Originally discovered as an intermediate during the synthesis of squaraine dyes, HfSQ dyes are synthetically versatile, exhibit high ε and have absorption maxima at ≈ 450–500 nm which is complimentary to the even more highly absorbing, fluorescent squaraines (λ_max_ ≈ 650 nm). So while HfSQ dyes absorb at similar wavelengths to Ru-bipy dyes and so can act as potential replacements, squaraine dyes absorb where Ru-bipy dyes are less sensitive [40]. Half-squaraines have been tested in ZnO devices giving η = 0.27% [40] and 0.53% [41] and 3.54% [42] in TiO_2_ devices. The synthetic versatility of HfSQs and squaraines has led to the publication of squaraines absorbing across the AM 1.5 solar spectrum, many of which efficiently harvesting light in the near infrared (NIR) [43–46]. This makes these dyes ideal candidates for co-sensitization, i.e. the use of more than a single dye as sensitizer in a single device.

### Theory

Modelling organic dye molecules in the gas-phase or adsorbed to a surface is the most straightforward aspect of modelling DSC devices, and is commonly done using DFT [47]. Potential issues can arise where the molecules contain heavy elements, in which case the pseudopotential might not exist. However, this can be addressed by, for example, generating them on-the-fly. Furthermore, for heavy elements spin orbit coupling needs to be included [47]. Modelling isolated dye molecules or dye-plus-anchor groups in the gas-phase enables calculation of their HOMO and LUMO levels [48], which can be combined with Langmuir adsorption isotherms to postulate the orientation of the dyes on the TiO_2_ surface [49].

Dye chromophores in themselves are highly amenable to computational modelling. Jacquemin et al. investigated the influence on the visible spectra of thioindigo dyes caused by small modifications [50]. Even without statistical post-treatment they obtained quantitative predictions, as long as the methodology was of a high enough quality. As another example, Sánchez-de-Armas et al. used TD-DFT to study the electronic structure and optical properties of five coumarin dyes for use in DSC devices [51]. They successfully identified relevant criteria to predict the sensitization efficiency of these molecules; among them the LUMO energy with respect to the CB edge. As pointed out by Le Guennic and Jacquemin, careful attention needs to be paid to the modelling methodology and parameters to obtain robust and consistent results, even within a specific class of chromophores [52].

## Dye anchoring groups

### Experiment

**Figure 3.**
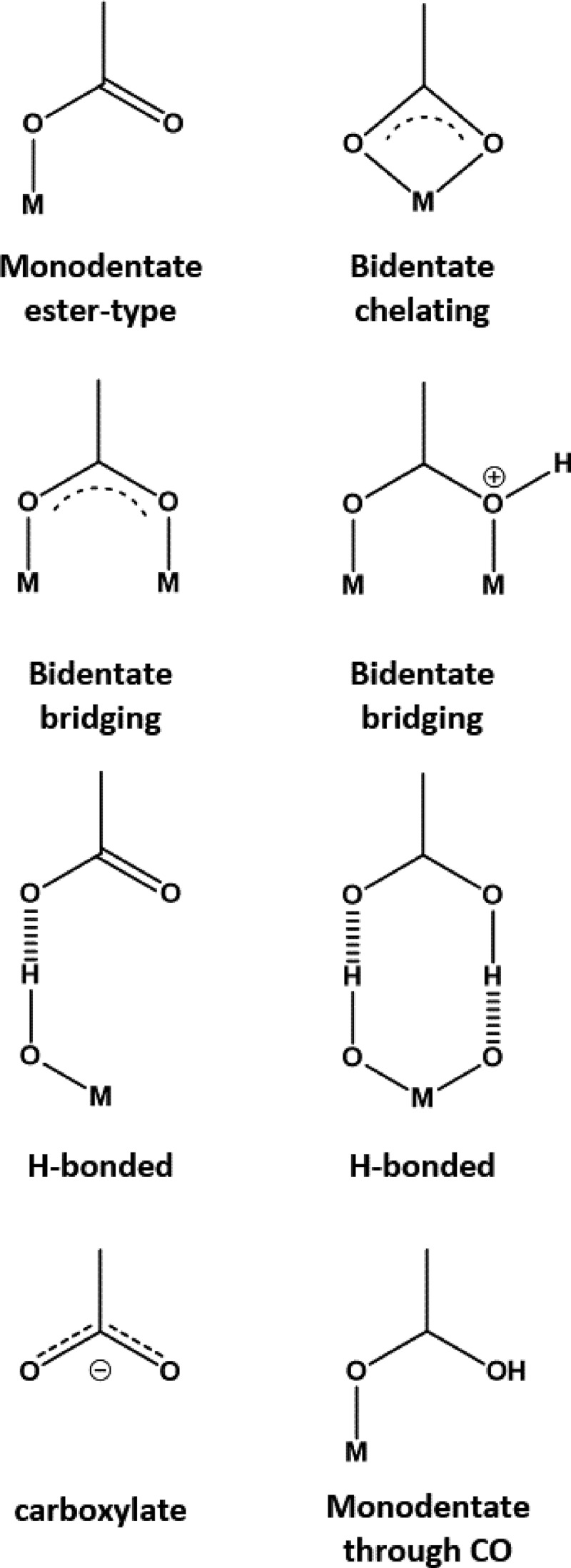
Possible binding modes for a carboxylate anchor group binding to a metal oxide surface. Reproduced with permission from [85].

Dye molecules can chemisorb to the metal oxide surface through strong covalent bonds, which have the most impact on how the dye anchors to the mesoporous metal oxide. The orientation of the dyes on the metal oxide surface is also affected by other (weaker) types of physisorption interactions (e.g. hydrogen bonding, electrostatic interaction, van der Waals forces, hydrophobic interactions or physical entrapment), surface packing and interaction with the electrolyte [53]. The exact fashion in which dye molecules adsorb to the surface will depend on the specific dye molecule and its anchoring group(s). Figure 3 displays 8 possibilities for the chemisorption of a single carboxylic acid (COOH) anchor to a metal oxide surface; this being the most widely used anchor group for DSC devices.

It is widely reported that anchoring groups deeply affect the performance of DSC devices and play a very important role in electron transfer. In addition to carboxylate, cyanoacrylic acid is the other most commonly-used DSC anchor group; both due to their electron-withdrawing effects. Carboxylic acids are most widely used in inorganic dyes where metal-to-ligand-charge-transfer (MLCT) transitions dominate. By comparison, cyanoacrylic acids are the most commonly used anchors in organic D−ᴨ−A dyes as these can increase the conjugation of the dye chromophore and therefore can extend the spectral response of the dye.

With the aim of more stable devices with higher efficiencies, many novel anchor groups have been investigated. These include; pyridine, phosphonic acids, tetracyanate, perylene dicarboxylic acid anhydride, 2-hydroxylbenzonitrile, 8-hydroxylquinoline, pyridine-N-oxide, hydroxylpyridium, catechol, hydroxamate, sulfonic acid, acetylacetanate, boronic acid, nitro, tetrazole, rhodanine, and salicylic acid substituents [53].

Of these examples, phosphonic acid anchoring groups have been reported to be extremely stable, desorbing from the photoelectrode 5–1000 times more slowly than carboxylic acid due to the stability of the P–O–M bonds. This, combined with the reduction in conjugation and the tetrahedral geometry of the phosphorous centre, ultimately limits its charge transfer adversely affecting the rate of electron injection [53]. Hydroxamic acids display a huge potential for use in DSC devices. Initially used in DSC devices by McNamara et al. [54], hydroxamic acids have proved to be extremely stable in water. A study by Crabtree et al. compared hydroxamic acid linkers directly with carboxylic and phosphonic acid [55]. Out of these three anchors, hydroxamic acid proved to be superior due to its higher photo-generated current densities. Inorganic dyes were used as the sensitizers in this study. However, commonly used organic dyes with carboxylic groups can be converted to hydroxamic anchors using a simple reaction. It is then possible to investigate the I-V and stability of these linker groups for organic dyes in DSC devices.

Boronic acids are another interesting and novel anchor group. Initially investigated due to their strong interaction with TiO_2_ [56], boronic acids show poor surface coverage with a single anchoring group which led to a poor incident photon conversion efficiency (IPCE) of 22%. However, when two boronic anchors were introduced this increased significantly to 60% indicating that two anchors gave a much better coverage on the photoelectrode surface. Unfortunately, the paper fails to report standard device efficiency testing suggesting ‘non-radiative deactivation from the excited sensitizer competes with charge injection’. This suggests that the devices had extremely low efficiencies. This could be down to the electron injection being too slow or slower than the deactivation. Although ‘low anchor performance’ is disappointing from a dye point of view, it could still prove useful for other areas of a device such as additives. By combining additives with a boronic acid anchor, these could be bound to the photoelectrode, potentially reducing dye aggregation without inhibiting their ability to suppress charge recombination.

The major problem when comparing and categorizing different anchoring groups is that they are often bound to very different chromophores, and it is difficult to separate the influence of one from the other. For the best performing devices, the HOMO/LUMO levels of the chromophore and anchor align, eliminating the internal losses of the device [7].

**Figure 4.**
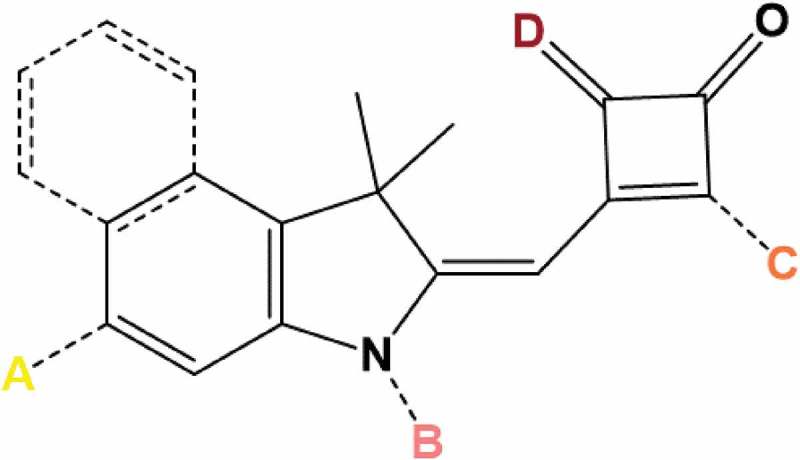
Dye anchoring points A, B, C and D on a half-squaraine chromophore. Reproduced with permission from [7].

**Figure 5.**
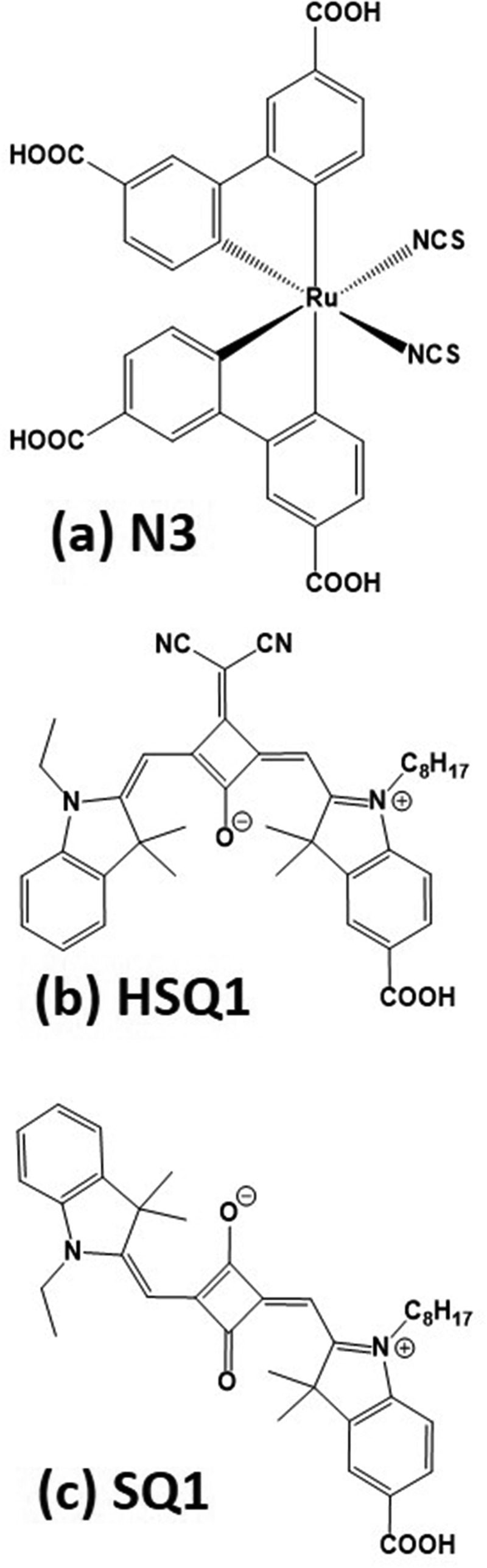
Molecular structures of (a) N3, (b) HSQ1 and (c) SQ1. Reproduced with permission from [68].

Figure 4 shows the core chromophore of half-squaraine (HfSQ) dyes along with points around the dye periphery which can be modified in a controlled way with different anchor groups [7,8]. In this way, HfSQ dyes can be used as sensitizers not only in their own right, but also as a model chromophore to predict similar effects in other dyes. This allows us to study the effects of different modifications to the chromophore and what those effects do to device performance. A paper published in 2014 by Holliman et al. looked at using a HfSQ chromophore to study the effects of the anchoring group position on DSC performance. It is generally believed for the best performing dyes that a D-ᴨ-A-anchor system must be chemisorbed with the acceptor unit situated closest to the metal oxide surface. Therefore, in the case of the half-squaraines, the best performing device should have an anchor on the squaric acid unit, as this is the acceptor. Several dyes were made each with the carboxylic anchoring group on a different segment of the dye, see Figure 4. One dye linked via the squaric acid, positions C and D in Figure 5 (Acceptor/LUMO), another had its anchor on the indole, position A (Donor/HOMO) and the third dye had an anchor coming off the nitrogen of the indole, position B (between HOMO and LUMO). As expected, the best performing dye linked via the squaric acid unit. The increase in performance was due to an increase in *V*_oc_ [7]. A follow up paper by Holliman et al. [8] looked at how multiple linkers affected the orientation of the dye on TiO_2_ surfaces. When dyes with a single anchoring group were bonded to the TiO_2_ they must bind at the only site available to them; the best anchoring group being located at the squaric acid moiety. When a second anchoring group was introduced (off the N of the indole), little effect was seen on the dye HOMO–LUMO gap. However, the data showed a significant increase in device stability and efficiency, achieving the highest ever recorded efficiency [8] for a HfSQ device. Devices could be measured over days rather than minutes suggesting that the dye was bound to the titania via two anchoring groups. Further analysis showed that, although modifying the squaric acid moiety of HfSQ with a vinyl dicyano group causes favourable broadening and bathochromic shift of the absorption peak, poor FF and *V*_oc_ were due to dye desorption when devices were filled with liquid electrolyte. The authors ascribed this to a stronger interaction of the nitrile groups on the dye with the acetonitrile solvent used to make the electrolyte compared to weaker physisorption interactions with the metal oxide surface. The next dye introduced an anchoring group bound to the indole benzene. When this dye was adsorbed onto titania a large bathochromic shift was observed. This, alongside very similar device performance, suggested the dye was bound through the anchoring group on the squaric acid unit rather than through the benzene indole. Finally, a dye with three anchoring groups was tested. A significant decrease in ε was observed, leading to a drop in device performance. Together with the research of Wu et al. [57] who studied TPA-based dyes with corhodanine acceptor/anchor units (Table 1), this research suggests two anchors can have a positive impact on device performance by improving *J*_sc_, whereas three anchor groups had a negative impact.

**Table 1.** DSC device parameters for triphenylamine dyes with 1,2 or 3 linkers. Errors in brackets. Reproduced with permission from [57].

	TPACR1	TPACR2	TPACR3
*J*_sc_/mA cm^−2^	12.94 (0.03)	15.03 (0.06)	9.37 (0.04)
*V*_oc_/V	0.52 (0.01)	0.55 (0.02)	0.41 (0.01)
FF	0.67 (0.01)	0.63 (0.01)	0.68 (0.00)
η/%	4.59 (0.01)	5.30 (0.01)	2.61 (0.01)

**Figure 6.**
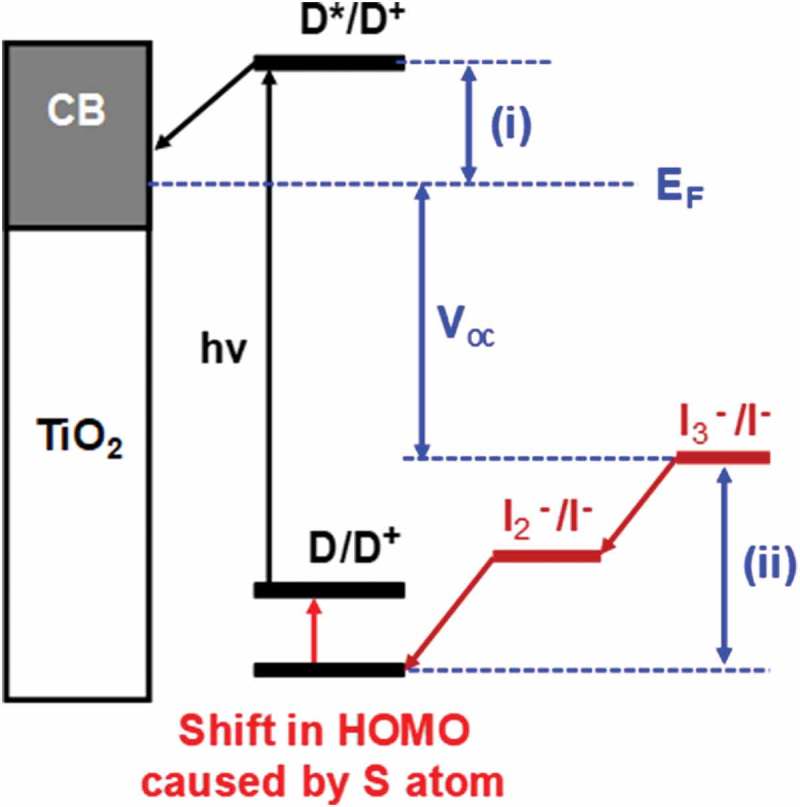
Shift in HOMO caused by the addition of sulphur atom to half-squaraine sensitizer. EF = Fermi level, (i) dye injection overpotential and (ii) dye regeneration overpotential. D = ground state dye, D* = excited state dye, D^+^; = oxidized dye, CB =conduction band, hν = sunlight and V_oc_ = open circuit voltage.

Wu et al. report that, if the functionality of the molecule allows, TPA dyes preferentially bind through the acceptor unit, in the order D-ᴨ A-anchor. They also report that, if a second anchor on the dye is close enough to the photoelectrode, it will also bind thereby improving the device stability. However, they also suggest that, if the dye has a third anchor (or a second far from the main binding site) then it is believed to not take part in the electron injection process, but to act as a pathway for recombination.

### Theory

The potential effects of anchoring groups on device efficiency have been explored computationally using DFT. Insight has been gained from the HOMO/LUMO levels, bridging, and orientation of the anchoring groups at TiO_2_ surfaces [58], as well as the relative stability of adsorption modes [59]. For example, carboxylic acid anchoring groups predominantly adsorb via bidentate bridging [60–62] with a predicted stability greater for the bidentate than the monodentate. However, the dominant adsorption mode changes on surface hydration [63] and surface planarity or curvature [64]. These model-dependent differences in the results highlight the importance of the system set-up, and the need of the modeller to gain a thorough understanding of the experimental system under scrutiny.

## Dye loading and co-sensitization

### Experiment

Durrant et al. first reported a step-wise approach to co-sensitization by first sorbing a Ru-bipyridyl dye onto TiO_2_, followed by treatment with aluminium isopropoxide to deposit an Al_2_O_3_ layer before sorbing a Ru-phthalocyanine dye [65]. Up until recently, the timescale for co-sensitization was time-consuming taking many hours and also unreliable in that dye loadings were hard to control and even harder to reproduce. The process needed to be sped up considerably in order to reduce costs, to make DSC devices a more scaleable PV technology and to improve the reproducibility of the co-sensitization process. In 2010, Holliman et al. reported the first ultrafast co-sensitization, with the process taking < 5 min [17] with ultra-fast tri-sensitization reported in 2012 [17]. As NIR sensitizers, squaraines could be used in conjunction with Ru-bipy dyes to harvest more light and therefore improve device efficiency [66]. By use of several dyes, co-sensitization has led to the highest ever performing DSC device with η = 14.7% [67].

Dye loading and processing during the co-sensitization of different dyes can be difficult, as partition coefficient (*K*_d_) and molar extinction coefficient (ε) can vary enormously between dyes. One way to address this is to consider how dyes can self-assemble at the metal oxide surface, because dye loading and surface organization is crucial in a multi-dye system. Thus, in a single-dye solution, a low *K*_d_ value is usually addressed by dyeing for longer and rinsing away the excess. However, this is not suitable for a co-sensitized system where if one dye is preferentially adsorbed over another, it can therefore be very difficult to increase the loading of the lower *K*_d_ dye. This can be equally problematic for dyes even with slightly different partition coefficients. But adsorption problems can be overcome and dye loadings can be controlled within the system if self-assembly is used to control the competitive sorption process of two or more dyes.

In addition, in terms of optimizing the spectral response, the co-sensitization process is not as simple as mixing two or more dyes together and expecting an increase. It is also important to consider matching the spectral response of the dyes. Having two dyes absorbing the same wavelengths effectively means the dyes are competing for the same photons, leading to low efficiencies and low *J*_sc_. However, two dyes absorbing in different areas of the AM 1.5 solar spectrum can act like a single panchromatic dye, absorbing more photons and producing a higher *J*_sc_ and therefore a higher η [7,8,17,67]. However, the HOMO/LUMO energy levels (of the different dyes) need to differ sufficiently (> 0.2 eV) from one another to inhibit any electronic interactions between them. Any interaction between the energy levels provide alternative (dye-to-dye) electron pathways leading to a decrease in device efficiency.

The physical interactions between the different dyes also need to be understood and carefully controlled in order to decrease dye aggregation because when multiple layers of dyes are present, the outer layer of dye interacts with light first. If this outer layer absorbs light, it does not inject the resulting excited electrons into the TiO_2_ because it is not electrically connected to the TiO_2_. At the same time the inner layer cannot absorb as much light because it is effectively being filtered by the outer layer. So dye aggregation can drastically decrease DSC device efficiency. However, it can be overcome by changing the dye structure, for example by including long, bulky, non-polar alkyl chains and aromatic groups into the dye structures which decrease inter-molecular interactions between the dye molecules. Another approach is to change the configurational structure of the dye to inhibit aggregation. For example, by changing the *cis*-squaraine SQ1 dye into the *trans*-squaraine HSQ1 dye, Qin et al. [68] found a significant improvement in device efficiency for the N3/HSQ1 combination over that of N3/SQ1 (Figure 5). Although these approaches can reduce aggregation and inhibit electron transfer between the dyes, they can also reduce the amount of dye on the photoelectrode, requiring careful control of the size of the functional groups. Adding bulky groups to dyes also has the indirect effect of making the interrogation of dye structures more difficult because it makes it more difficult to grow single crystals suitable for X-ray structural analysis. This has a knock-on effect to the theoretical studies because single-crystal data are a useful starting point for inputting data into molecular simulations.

### Theory

Gaining insight from computational modelling significantly contributes to addressing the co-sensitization issues mentioned above. For example, using both DFT and TD-DFT, Kusama et al. identified that cyclic bonding between the peripheral carboxyl groups of a Ru-complex and organic dye was responsible for increasing sensitization and inhibiting dye aggregation [69]. Pastore and De Angelis have also provided a comprehensive overview to modelling intermolecular interactions in DSC devices including co-sensitization using DFT, TD-DFT, and Møller Plesset (e.g. MP2) to account for weak dispersive interactions [70]. They describe a stepwise process of simulating the DSC components individually, then progressing to exploring the interactions between pairs of the component parts of the DSC devices. For example, they explore dye aggregation and co-sensitization by first simulating a single dye adsorbed to the TiO_2_ surface. They then include subsequent dyes based on the relative strengths of adsorption on to TiO_2_ and intermolecular interactions between dye molecules. Using this approach, a convincing DSC model, including accurately reproducible experimental fluorescence resonance energy transfer (FRET) kinetics was developed [71].

The computational modelling work of Tateyama et al. [66] was used to identify the improved efficiency reported in the work of Qin et al. [68], where *trans*-squaraine SQ1 co-sensitized with the Ru-bipy N3 dye was found to be more effective than the equivalent *cis*-squaraine (Table 1). It was reported that just a single hydrogen bond between SQ1 and N3 caused SQ1 to hinder the regeneration of N3. Tateyama et al. also found that the steric hindrance of the *cis* isomer inhibited the interaction with N3 leading to an improvement of IPCE compared to either N3/SQ1 or neat N3.

## Interfaces

### Experiment

DSC devices are multi-layer, electronic devices which contain very different chemical components which meet at several key interfaces (e.g. organic dye molecules interfaced with metal oxide nanoparticles and a charge carrying electrolyte interfacing with a dye layer). The difficulty in using experimental methods to studying these interfaces is that they are all effectively connected to each other in series so one challenge is to separate out the signals from each interface. One method to do this is impedance spectroscopy which can measure the electrical characteristics associated with the different interfaces [72]. Another challenge is that, because mesoporous TiO_2_ films are used for the photoanodes, there are effectively thousands of interfaces in a DSC device. This means that, although it is possible for instance to use transient photocurrent and photovoltage decay data to study photoelectrode sintering [16], what is observed is actually an average for the whole device. By comparison, modelling approaches can study detailed interfacial processes and can, for example, change one or two atoms in a model and observe the effect. This would be very difficult to do experimentally to obtain statistically meaningful data.

### Theory

De Angelis [47] describes the modelling interfaces (of DSC devices) as ‘… the most difficult task for hybrid/organic photovoltaics modelling, due to the inherent complexity of the investigated systems’. For example, if we consider liquid DSC devices, then the region of electrolyte immediately adjacent to the metal oxide surface will behave differently to that of the bulk electrolyte which is further away from the surface. This electrolyte-surface region was originally described as the ‘electric double layer’. In the early 2000s, techniques such as the MUSIC method (MultiSIte Complexation) were used alongside experimental results to determine the position of cations and the electrolyte with respect to the surface. It was sufficiently sophisticated to include the effects of temperature [73], and eventually interactions within the electric double layer itself [74].

A second interfacial region is that between the dye/sensitizer and the TiO_2_ surface. The computational modeller needs to know how many dye/sensitizer molecules cover the surface, how they attach to the surface, and how they orient themselves with respect to this surface. O’Regan and Durrant [18] estimate (for a typical DSC photoelectrode material) there are ~ 600 dye molecules on the surface of an 18 nm-sized particle of TiO_2_. If each dye molecule contains 100’s of atoms and the TiO_2_ model is represented by 10,000’s of atoms, then the resulting model is of the order of 100,000’s of atoms. This clearly impacts on computational time. To identify local minima that determine the orientation of the dye molecules with respect to the surface, MD can be used. However, although the size of the system is manageable for MD, even using modest computational resources, the major obstacle is the availability of a force field that captures the interactions inherent in both the inorganic TiO_2_ and the organic dye. If there is no appropriate force field combining the inorganic and organic components, an option is to tailor-make one, but this is a lengthy and complex process involving empirical data and/or QM and *ab initio* MD [75]. Another option is to decrease the number of molecules by at least one order of magnitude and employ *ab initio* MD such as Car-Parrinello MD [76].

## Outlook

To satisfy lifetime requirements for solar cell technology, there is a shift from DSC devices with liquid electrolytes to those using solid state HTM electrolytes. These have been much less studied than their liquid counterparts but, with correctly aligned energy levels and the right degree of interfacial control, there is great potential for them to out-perform liquid DSC devices. In addition, while the 20+ years of liquid DSC research has provided a wide range of effective light harvesting dyes, these have been optimised for liquid electrolytes and there is a clear need to use theory and experimentation to better understand the dye-electrolyte interface when using solid, HTM charge carriers rather than liquid electrolytes. In this context, future work should link experiment and theory more closely together by using ‘atomic tags’ (e.g. sulphur atoms) into a known chromophore (e.g. a half squaraine) to study electron injection dynamics and dye orientation at TiO_2_ surfaces using angle-resolved X-ray photoelectron spectroscopy (AR-XPS). These data could then be used in conjunction with DFT calculations of dye energy levels (e.g. HOMO and LUMO) and preferred dye orientations on model TiO_2_ surfaces.

In support of the importance of the dye-oxide interface, it has been suggested in multiple papers [77–79] that sulphur can play an important role in the electron transfer between the dye and the electrolyte. Some papers have claimed that placing a sulphur atom in the outermost region of the dye (HOMO) exposes the orbitals to the I^−^/I_3_^−^ electrolyte which should aid regeneration of the oxidised dye by the electrolyte redox couple. However, other papers claim that sulphur can have a negative influence on the performance of a device. They state, if the sulphur atom is in the LUMO of the dye it can provide binding sites for oxidised species in the redox shuttle (I_2_ or I_3_^−^), increasing their concentration close to the TiO_2_ surface and thus accelerating the recombination process [77–79]. Hence, a sulphur ‘atomic tag’ could provide a useful probe linking experiment and theory together.

A 2007 review of modelling electron-injection dynamics concluded with seven summary points, one of which stated that ‘The average behaviour … is relatively independent of these specifics and can be predicted from a small number of concepts …’ [80]. With the increasing availability of more complex computational techniques, and the accumulated experience that modelling artefacts might have influenced results, it remains to be seen whether the same seven points are applicable in 2018. Previously overlooked detail such as the influence of the electrolyte and solvent molecules on device efficiency [81] might prove to be the source of future computational and experimental breakthroughs.

From a theoretical perspective the development of predictive methods to model DSCs is an on-going process. For example, a method to predict charge injection rates in DSC devices whereby they can be deconstructed into their three components – TiO_2_, dye and HTM – and the sub-systems connected by a (basis set) coupling matrix has been reported and used to predict the charge injection rate and hence screen chromophores using a desktop computer [82]. Solid state DSC devices are also being designed using DFT, TD-DFT- including PCM – on clusters of TiO_2_ nanoparticles [83]. Data analytics also has a role to play in the development and improvement of DSC devices. For example, large-scale data mining techniques encoding ‘… molecular design rules.’ have discovered a new class of dyes for DSC applications [84], and are laying the foundations of predicting the composition of DSC devices.

## Conclusions

The strength of experimental work on DSC devices lies in the demonstration of optimized devices with all the sub-components operating simultaneously under normal working conditions. In addition, experimental work enables the scale-out of devices in terms of making larger devices or testing them for much longer to measure device lifetime. It is also possible to study different sub-components of DSC devices although this will tend to give an average of what is happening within that whole component. By comparison, the strength of theoretical modelling is that it can study even very small changes in the sub-component of a device (e.g. changing a single atom) in a very controlled way. Modelling can also focus on very specific parts of a device which is much more difficult experimentally. Ultimately, the real benefit is achieved when experiment and theory are used together in an iterative approach where each informs the other.
